# Association of daily physical activity with brain volumes and cervical spinal cord areas in multiple sclerosis

**DOI:** 10.1177/13524585221143726

**Published:** 2022-12-27

**Authors:** Valerie J Block, Shuiting Cheng, Jeremy Juwono, Richard Cuneo, Gina Kirkish, Amber M Alexander, Mahir Khan, Amit Akula, Eduardo Caverzasi, Nico Papinutto, William A Stern, Mark J Pletcher, Gregory M Marcus, Jeffrey E Olgin, Stephen L Hauser, Jeffrey M Gelfand, Riley Bove, Bruce AC Cree, Roland G Henry

**Affiliations:** UCSF Weill Institute for Neurosciences, Department of Neurology, University of California San Francisco, San Francisco, CA, USA/Department of Physical Therapy and Rehabilitation Science, University of California San Francisco, San Francisco, CA, USA; UCSF Weill Institute for Neurosciences, Department of Neurology, University of California San Francisco, San Francisco, CA, USA; UCSF Weill Institute for Neurosciences, Department of Neurology, University of California San Francisco, San Francisco, CA, USA; UCSF Weill Institute for Neurosciences, Department of Neurology, University of California San Francisco, San Francisco, CA, USA; UCSF Weill Institute for Neurosciences, Department of Neurology, University of California San Francisco, San Francisco, CA, USA; UCSF Weill Institute for Neurosciences, Department of Neurology, University of California San Francisco, San Francisco, CA, USA; UCSF Weill Institute for Neurosciences, Department of Neurology, University of California San Francisco, San Francisco, CA, USA; UCSF Weill Institute for Neurosciences, Department of Neurology, University of California San Francisco, San Francisco, CA, USA; UCSF Weill Institute for Neurosciences, Department of Neurology, University of California San Francisco, San Francisco, CA, USA/Department of Brain and Behavioral Sciences, University of Pavia, Pavia, Italy; UCSF Weill Institute for Neurosciences, Department of Neurology, University of California San Francisco, San Francisco, CA, USA; UCSF Weill Institute for Neurosciences, Department of Neurology, University of California San Francisco, San Francisco, CA, USA; Department of Epidemiology and Biostatistics, University of California San Francisco, San Francisco, CA, USA/Department of Medicine, University of California San Francisco, San Francisco, CA, USA; Department of Epidemiology and Biostatistics, University of California San Francisco, San Francisco, CA, USA; Department of Epidemiology and Biostatistics, University of California San Francisco, San Francisco, CA, USA; UCSF Weill Institute for Neurosciences, Department of Neurology, University of California San Francisco, San Francisco, CA, USA; UCSF Weill Institute for Neurosciences, Department of Neurology, University of California San Francisco, San Francisco, CA, USA; UCSF Weill Institute for Neurosciences, Department of Neurology, University of California San Francisco, San Francisco, CA, USA; UCSF Weill Institute for Neurosciences, Department of Neurology, University of California San Francisco, San Francisco, CA, USA; UCSF Weill Institute for Neurosciences, Department of Neurology, University of California San Francisco, San Francisco, CA, USA/Department of Radiology, University of California San Francisco, San Francisco, CA, USA

**Keywords:** Multiple sclerosis, Fitbit, remote monitoring, activity level, spinal cord gray matter area, cervical MRI, brain MRI

## Abstract

**Background:**

Remote activity monitoring has the potential to evaluate real-world, motor function, and disability at home. The relationships of daily physical activity with spinal cord white matter and gray matter (GM) areas, multiple sclerosis (MS) disability and leg function, are unknown.

**Objective:**

Evaluate the association of structural central nervous system pathology with ambulatory disability.

**Methods:**

Fifty adults with progressive or relapsing MS with motor disability who could walk >2 minutes were assessed using clinician-evaluated, patient-reported outcomes, and quantitative brain and spinal cord magnetic resonance imaging (MRI) measures. Fitbit Flex2, worn on the non-dominant wrist, remotely assessed activity over 30 days. Univariate and multivariate analyses were performed to assess correlations between physical activity and other disability metrics.

**Results:**

Mean age was 53.3 years and median Expanded Disability Status Scale (EDSS) was 4.0. Average daily step counts (STEPS) were highly correlated with EDSS and walking measures. Greater STEPS were significantly correlated with greater C2-C3 spinal cord GM areas (ρ = 0.39, *p* = 0.04), total cord area (TCA; ρ = 0.35, *p* = 0.04), and cortical GM volume (ρ = 0.32, *p* = 0.04).

**Conclusion:**

These results provide preliminary evidence that spinal cord GM area is a neuroanatomical substrate associated with STEPS. STEPS could serve as a proxy to alert clinicians and researchers to possible changes in structural nervous system pathology.

## Introduction

In early multiple sclerosis (MS), atrophy and focal lesion load in the spinal cord have important implications for prognosis and diagnosis.^1^ Furthermore, spinal cord atrophy predicts the time to relapse-free disability worsening and secondary progressive MS in relapsing remitting MS.^2^ Later in the disease, and in people with progressive forms of MS, spinal cord atrophy correlates with standard clinical disability metrics (i.e. the Expanded Disability Status Scale (EDSS)) as well as disability progression.^3–6^

Until recently, the relative contributions of spinal cord white matter (WM) and gray matter (GM) pathology were unattainable in vivo. However, the use of phase-sensitive inversion recovery (PSIR) imaging^7^ can characterize lesions, estimating total cord areas (TCA),^8^ GM areas, and WM areas, using practical scanning times of <2 minutes per level.^9–11^ In MS, cervical and thoracic spinal cord GM atrophy correlated with disability and function of the upper and lower limbs.^4,5^ In addition to spinal cord atrophy, deep GM atrophy of the brain (including thalamus) and cortical atrophy are associated with MS disability and motor function.^12,13^

Remote activity monitoring has many advantages in evaluating everyday physical and motor function and disability in neurological populations.^14^ Remote monitoring outcomes are continuous, ecologically valid, and sensitive to change over time, providing realistic, relevant measures of how a person is performing outside of the clinic setting.^14,15^ Daily step count (STEPS) was shown to detect disability change over 1 year, even when conventional measures (e.g. EDSS and walking speed) remained stable. Furthermore, studies on remote activity monitoring showed that relatively small sample sizes were needed to detect a clinically meaningful difference in STEPS between treatment groups.^16,17^

The objective of this study was to evaluate the association of structural central nervous system (CNS) pathology (MR imaging) with physical ambulatory disability (STEPS). The primary hypothesis was that spinal cord atrophy is a relevant anatomic substrate for STEPS, and therefore, spinal cord areas as well as brain deep and cortical GM volumes would correlate with STEPS.

## Methods

### Cohort recruitment and study design

Fifty-two participants with MS were recruited into this prospective, observational cohort study if they were enrolled in either the University of California, San Francisco (UCSF) EPIC study^18^ or Neuronal Determinants of Motor Disability in MS (MOTOR) study.^19^ Inclusion and exclusion criteria are detailed in Table 1.

**Table 1.** Inclusion and exclusion criteria.

Inclusion for fitMRI	Exclusion for fitMRI
● Male or female, over 18 years ● Clinical diagnosis of MS (either relapsing or progressive; as per 2010 International Panel criteria^24^) ● No relapse in the 3 months prior to study entry ● Could walk at least 2 minutes (with or without an assistive device) ● Access to Wi-Fi in their home or community	● No cardiovascular or musculoskeletal comorbidities affecting gait.
Inclusion for MOTOR study	Exclusion for MOTOR study
Evidence of asymmetric motor or symmetric disability in the neurological examination attributed to MS as follows: ● Difference in muscle strength of >1 point on the Medical research Council Muscle Strength Grading System between right and left side in distal and/or proximal muscle groups of at least one limb AND (either hyperreflexia of deep tendon reflexes AND/OR another positive pyramidal sign (e. g. asymmetric silent or positive Babinski sign, diminished cutaneous abdominal reflexes) on the affected side). ● OR (bilateral muscle weakness (as measured by MRC-Grading)) AND (asymmetry in functional motor tests in the EDSS (hopping, tapping, heel and toe walking)) AND (asymmetry of the deep tendon reflexes with hyperreflexia on the more affected side AND asymmetric silent or positive Babinski sign in the limb more affected in the functional tests).	● Any other orthopedic, medical, or neurological diagnosis contributing to asymmetric weakness in the lower limbs. ● Contraindications for MRI: metal implants or non-removable metallic objects not fixed to the skeleton or implants controlled by physiological signals such as pacemakers, implantable cardioverter defibrillators, and vagus nerve stimulators.
Inclusion for EPIC study	Exclusion for EPIC study
● Persons aged 18–70 years. ● A diagnosis of MS (Thompson et al., 2018),^25^ with dissemination in time and space. A small number of patients with CIS may also be included if they fulfill three of the four Barkhof criteria for dissemination in space as per application of the McDonald criteria (Thompson et al., 2018).^25^ ● No relapse within the 4 weeks prior to screening. ● EDSS of between 0 and 7.5. ● Willingness to return each 12 months for the follow-up and MRI and blood draw. ● Able and willing to sign an informed consent.	● Subjects receiving corticosteroids for any reason within 30 days of screening. If non-systemic steroids are being used for other chronic inflammatory conditions, subjects may be included at the discretion of the investigator. ● Subjects participating in ongoing MS clinical trials with non-approved drugs. ● Recent history or suspicion of current drug abuse or alcohol abuse within the last 6 months. ● Any concurrent illness, disability, or clinically significant abnormality (including laboratory tests) that may prevent the subject from safely completing the assessments required by the protocol. ● Unable to give consent. ● For neuroimaging studies additional exclusion criteria are: ● Individuals who are unable to undergo an MRI due to metal implants. ● Women who are pregnant.

MRI: magnetic resonance imaging; MS: multiple sclerosis; MOTOR Study: Neuronal Determinants of Motor Disability in MS; EDSS: Expanded Disability Status Scale; MRC: Medical Research Council.

**Figure 1.**
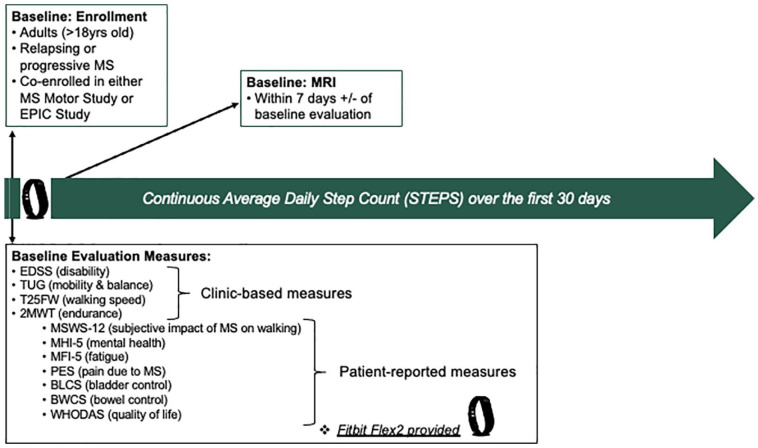
Study design—outline. EDSS: Expanded Disability Status Scale; TUG: Timed-Up and Go; T25FW: Timed 25-Foot Walk; 2MWT: 2-Minute Walk Test; MSWS-12: 12-item MS Walking Scale; MHI-5: 5-item Mental Health Inventory; MFIS-5: 5-item Modified Fatigue Index Scale; PES: Pain Effects Scale; BLCS: Bladder Control Scale; BWCS: Bowel Control Scale; WHODAS: WHO Disability Assessment Schedule; MRI: magnetic resonance imaging.

Participants were provided with a Fitbit Flex2 and were taught to set up and maintain (charge and sync) their devices. Participants were asked to wear the devices on their non-dominant wrist as much as possible for 30 days. Step count data were collected and stored securely using the Eureka Research Platform (https://info.eurekaplatform.org/) at the University of California, San Francisco (UCSF). Study team members were available for any questions regarding the Fitbit and replaced any lost devices within 36 hours. Figure 1 summarizes the study protocol (fitMRI study). The study was approved by the UCSF Institutional Review Board, and all participants provided written consent.

### Clinical and patient-reported assessments

At study entry, participants were evaluated by a neurologist for MS disability (EDSS) and by a physical therapist for mobility (Timed-Up and Go; TUG), walking speed (Timed 25-Foot Walk test; T25FW), and endurance (2-Minute Walk Test; 2MWT). Questionnaires (patient-reported outcomes; PROs) assessing common symptoms of MS were sent via secure email to the participants to be completed within 7 days of their initial evaluation. PROs included the 12-item MS Walking Scale (MSWS-12), 5-item Mental Health Inventory (MHI-5), 5-item Modified Fatigue Index Scale (MFIS-5), Bladder Control Scale (BLCS), and among others (details in Figure 1). Demographics including disease type (MS type), disease-modifying treatment (DMT), and body mass index (BMI) were recorded at the time of evaluation or retrieved from the patients’ electronic medical record. For analysis, participants were categorized into higher (>27 kg/m^2^) or lower (⩽27 kg/m^2^) BMI groups due to effects of obesity on gait and reported correlations of lower physical activity and higher BMI in society.^20^

### Image acquisition and analysis

Quantitative MRI was evaluated ±2 weeks from the study entry neurological evaluation. All participants were scanned on a Siemens 3T Skyra scanner with 64-channel head and neck coil and 32-channel spine coil.^5^ PSIR images were acquired at the C2-3 intervertebral disk level of the spinal cord. Brain imaging sequences acquired included magnetization prepared rapid acquisition gradient echo (MPRAGE) and fluid-attenuated inversion recovery (FLAIR) sequences.

**Figure 2.**
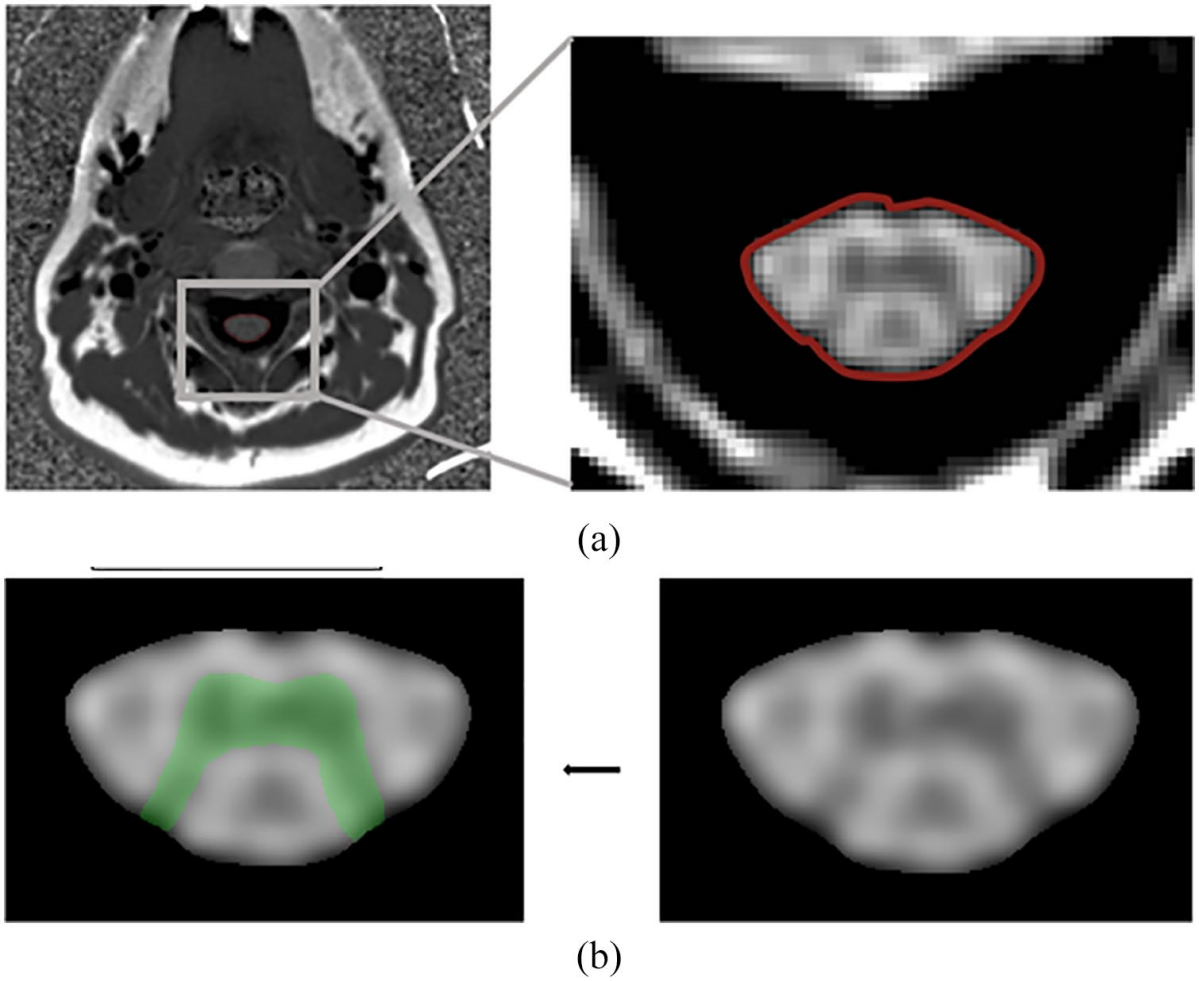
Example of cervical spinal cord area imaging techniques. (a) Axial slice of PSIR C2-C3. Axial slice of a one-dimensional PSIR C2-C3 spinal cord image. The cord is segmented using automated method and the border is shown delineated here in red. (b) Gray matter segmentation. The spinal cord is cropped and up sampled 10 times on all dimensions. The gray matter segmentation is then performed on this processed image. The resulting segmentation is shown in green. PSIR: phase-sensitive inversion recovery.

Total spinal cord area (TCA (C2-3)) and the spinal cord GM area were estimated from the PSIR images using fully automated methods of extraction (Figure 2).^10,21,22^ For all the PSIR acquisitions, the TCA were obtained using a convolutional neural network (CNN) trained on segmentations derived from the semi-automated JIM software (version 7.0, Xinapse Systems Ltd, West Bergholt, United Kingdom, http://www.xinapse.com). All GM areas were estimated using a two-step registration and local intensity–based segmentation method. Both methods were developed in the Henry Lab at UCSF (in-house software (unpublished) validated compared to manual tracings with high correlation). WM was calculated as the difference between TCA and GM. Angle correction was applied to all spinal cord outcomes via three-dimensional (3D) rotations using angles obtained from reference sagittal T2-weighted images.

Normalized brain GM and WM volumes were calculated using the FreeSurfer image analysis suite (available at http://surfer.nmr.mgh.harvard.edu/).^23^ Due to inter-participant differences in head size and therefore cranial volume, all MRI metrics were normalized to volume fractions using the total intracranial volume (skull volume) on an individual basis.^10,22^ All scans were reviewed by an experienced radiologist (EC) for new lesions.

### STEPS quality control

All participant activity data (STEPS) were downloaded from the secure Eureka platform. For quality control, days with <300 STEPS (indicating a potential lack of full day wear), and weeks with <3 days of activity were excluded from the analysis.^16^

### Statistical analyses

Histograms were generated to assess for normality of the data distribution. Metrics were transformed toward normal distributions to achieve normally distributed residuals in the regressions, reduce heteroskedasticity, and provide better confidence intervals. STEPS were skewed toward the fewer steps per day and were therefore transformed using the cube root. TUG and T25FW were transformed using the inverse score as previously described.^16^

#### Primary analysis

To evaluate the strength of correlation between STEPS and MRI quantitative metrics (spinal cord GM area, TCA, spinal cord WM area, brain cortical GM volume, brain deep GM volume, brain WM volume, total brain volume), Spearman’s rank correlation coefficient was performed for each outcome individually with a false discovery rate (FDR) correction to account for multiple comparisons.

To build a multivariable model of STEPS including MRI variables listed above, variable selection was performed using the least absolute shrinkage and selection operator (LASSO) including covariates (i.e. sex, age at disease onset, and disease duration) to determine the independent contributors of STEPS. Due to collinearities between cord measures, separate models were run with STEPS and (a) spinal cord GM and brain metrics, (b) spinal cord WM and brain metrics, and (c) TCA and brain metrics. The double LASSO procedure implemented in JMP was used, which performs both model selection and parameter shrinkage. For sensitivity, this analysis was repeated with EDSS as the response variable and the addition of STEPS as an explanatory variable.

Treatment effect was evaluated by including categorized DMT group (either platform therapy, high potency therapy or untreated) into the multivariable models. MS type was separately added into the model to assess how this term modified the relationships between STEPS and MRI.

A *p-*value of 0.05 was considered significant. All statistical analyses and figure generations were performed using JMP Pro 16 (SAS Institute, Cary, NC, USA; www.jmp.com). All reported *p*-values are FDR corrected.

## Results

### Participant characteristics

Fifty participants with MS, ages 24–77 years (mean = 53, standard deviation (SD) = 13.4), were recruited. The majority were women (66%) with relapsing MS (56%) and moderate disability (EDSS median = 4.0, interquartile range (IQR) = 2.5, range = 0.0–6.5). The median disease duration was 17.5 years (IQR = 23, range = 0–50). One-third (30%) were untreated at the time of initial evaluation. On average, over 30 days, participants took 5408 steps per day (SD = 3350) (Table 2). All participants provided step count data for the first 30 days. Two participants withdrew from the study in the first week due to personal issues, unrelated to the study protocol.

**Table 2.** Clinical and demographic characteristics of fitMRI participants.

Cohort	
Sample size (*N*)	50
Sex (female; *N*, %)	33 (66%)
Age at baseline—years, mean (SD)	53 (13.4)
BMI, mean (SD)	25.0 (5.1)
Disease duration—years, median (IQR, range)	17.5 (23.0–50)
Disease-modifying therapy (*N*, %)	
High potency therapy	29 (58)
Platform therapy	6 (12)
No therapy	15 (30)
MS type (progressive; *N*, %)	22 (44%)
EDSS at baseline, median (IQR, range)	4.0 (2.50–6.5)
STEPS, mean (SD)	5.408 (3.350)

MRI: magnetic resonance imaging; N: sample size; SD: standard deviation; BMI: body mass index; IQR: interquartile range; MS: multiple sclerosis; EDSS: Expanded Disability Status Scale; STEPS: average daily step count (over the first 30 days of the study).

Disease-modifying therapy: *High Potency Therapy* included natalizumab, rituximab, ocrelizumab, fingolimod, and dimethyl fumarate; *Platform Therapy* included interferon (IFN) beta-1b, glatiramer acetate, and glatopa and teriflunomide.

### Physical activity associations: clinic-based and patient-reported measures

Remotely monitored physical activity over 30 days (STEPS) was highly correlated with disability (EDSS; ρ = −0.60, *p* < 0.01), and pyramidal (ρ = −0.57, *p* < 0.01), cerebellar (ρ = −0.40, *p* < 0.01) and bowel and bladder (ρ = −0.57, *p* < 0.01) functional scale scores. Lower STEPS were associated with longer TUG (ρ = −0.52, *p* < 0.01) and T25FW (ρ = −0.569, *p* < 0.01) times. More STEPS were correlated with greater endurance (longer distances walked during the 2MWT; ρ = 0.61, *p* < 0.01).

Greater STEPS were associated with a better patient-reported perception of walking impairment (MSWS-12; ρ = −0.70, *p* < 0.01), better quality of life (WHO Disability Assessment Schedule (WHODAS); ρ = −0.51, *p* < 0.01), and less bother from bladder (BLCS; ρ = −0.47, *p* < 0.01) and bowel (Bowel Control Scale (BWCS); ρ = −0.39, *p* = 0.02) control. Greater fatigue (MFIS-5; ρ = −0.40, *p* < 0.01) and MS-related pain (Pain Effects Scale (PES); ρ = −0.30, *p* = 0.02) were correlated with lower STEPS. Self-reported mental health (5-item Mental Health Inventory) was not correlated with STEPS (ρ = −0.05, *p* = 0.69).

### BMI and physical activity

No correlation was observed between STEPS and BMI (ρ = 0.18, *p* = 0.39), although there was larger variance in STEPS for people with lower BMI (3450 steps vs 3019 steps for higher BMI) that was not statistically significant (*p* = 0.70).

### Physical activity associations: quantitative MRI measures

**Figure 3.**
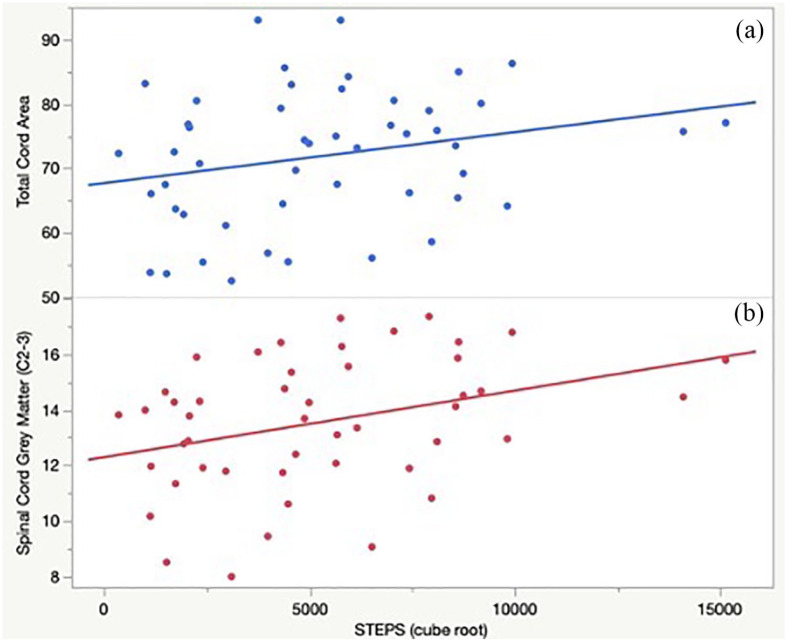
STEPS correlates with cervical spinal cord metrics in people with multiple sclerosis. (a) Greater total cord area (C2-C3) correlated with and greater STEPS (*p* = 0.01). (b) Greater spinal cord gray matter area (C2-C3) correlated with greater STEPS (*p* < 0.01). STEPS: average daily step count.

Univariate Spearman’s correlations between STEPS and MRI measures are shown in Table 3. After FDR correction, greater activity levels (higher STEPS) correlated significantly with greater spinal cord GM areas (C2-C3; ρ = 0.39, *p* = 0.04), greater cortical GM volume fraction (ρ = 0.32, *p* = 0.04), and greater TCA (ρ = 0.35, *p* = 0.04). Positive univariate trends toward greater STEPS and subcortical GM volume fraction (ρ = 0.37, *p* = 0.06) and brain volume (ρ = 0.31, *p* = 0.21) were found. Figure 3 illustrates univariate associations between normalized STEPS and quantitative spinal cord MRI measures.

**Table 3.** Spearman’s rank correlation between clinic-based, patient-reported, and quantitative MRI measures and average daily steps (STEPS).

Outcome	Spearman’s ρ	*p-*value
Clinic-based		
EDSS	−0.60	<0.01*
TUG	−0.52	<0.01*
T25FW	−0.60	<0.01*
2MWT	0.61	<0.01*
BMI	0.18	0.39
Patient reported		
MSWS-12	−0.70	<0.01*
MFIS-5	−0.40	<0.01*
MHI-5	−0.05	0.69
PES	−0.30	0.02*
BLCS	−0.47	<0.01*
BWCS	−0.39	0.02*
WHODAS	−0.51	<0.01*
Magnetic resonance imaging		
Spinal cord GM Area (C2-3)	0.39	0.04*
TCA (C2-3)	0.35	0.04*
Brain volume^a^	0.32	0.21
Subcortical GM volume^a^	0.37	0.06
Cortical volume fraction^a^	0.32	0.04*

MRI: magnetic resonance imaging; STEPS: average daily step count; EDSS: Expanded Disability Status Scale; TUG: Timed-Up and Go Test; T25FW: Timed 25-Foot Walk test; 2MWT: 2-Minute Walk Test; BMI: body mass index; MSWS-12: 12 item MS Walking Scale; MFIS-5: Modified Fatigue Impact Scale (5-item version); MHI-5: Mental Health Inventory (5-item version); PES: Pain Effects Scale; BLCS: Bladder Control Scale; BWCS: Bowel Control Scale; WHODAS: World Health Organization Disability Assessment Schedule (2.0); GM: gray matter; TCA: total cord area.

As expected, similar correlations were found with spinal cord white matter (which is calculated from “TCA—spinal cord GM”) and brain white matter (calculated from “total brain volume—brain GM”) and STEPS.

a Normalized for VScale.

* Statistically significant at *p* < 0.05 (after false discovery rate correction).

### Multivariable contributors of STEPS and disability

Multivariate models, using double LASSO, were performed with response variables (A) STEPS or (B) EDSS (disability) and separate models including GM model (brain and spinal cord GM), GM + WM model (total brain and cord), and a WM model (WM brain and spinal cord metrics). All models included sex, onset age, treatment type, and disease duration as covariates; STEPS was included as an independent variable in EDSS models.

(A) GM model: spinal cord GM (chi-square = 7.80, *p* < 0.01) was associated with STEPS; multivariate model *R*^2^ = 0.18. GM + WM model: only TCA (chi-square = 5.04, *p* = 0.02) was retained in the model (*R*^2^ = 0.14), and WM model: spinal cord WM was also significantly associated with STEPS (chi-square = 7.64, *p* < 0.01—full model *R*^2^ = 0.10).
(B) GM model: STEPS (chi-square = 18.84, *p* < 0.01) was a strong predictor of EDSS, removing GM MRI variables (full model *R*^2^ = 0.50). GM + WM model: STEPS (chi-square = 21.69, *p* < 0.01), TCA (chi-square = 4.72, *p* = 0.03), and onset age (chi-square = 4.48, *p* = 0.03) were retained as significantly associated with EDSS (full model *R*^2^ = 0.53), and in the last iteration including WM variables, WM model: STEPS (chi-square = 24.27, *p* < 0.01) and spinal cord WM (chi-square = 4.95, *p* = 0.03) were associated with EDSS (model *R*^2^ = 0.51).

## Discussion

These data reveal novel associations between objectively measured, daily physical activity, and cervical spinal cord (C2-3) GM area and TCA, indicating that people with MS who have smaller total cord and spinal cord GM areas tend to take fewer STEPS. The spinal cord GM area correlations build on documented associations between deep brain GM^26^ and in-clinic disability measure with remote activity monitoring outcomes (STEPS), suggesting that STEPS could be utilized as a proxy to alert clinicians and researchers about possible changes in structural nervous system pathology and worsening MS.

### Structural imaging relationships

Associations from MR imaging of the spinal cord may be particularly informative about mobility in people with MS.^27^ Brain volume and physical activity have been correlated with MS disability status.^13,16^ In healthy aging, physical activity has also been associated with brain GM volume.^28^ The effect of physical activity on CNS structures in MS is unknown, although higher levels of physical activity are hypothesized to have a beneficial effect on brain health.^29^ Moderate-to-vigorous physical activity was correlated with MRI whole brain GM, WM, and deep GM matter structures in a study monitoring 39 people with MS over 7 days using an ActiGraph (model GT3X + research accelerometer).^27^ In the same study, no correlation was found between brain MRI metrics and people who performed low levels of physical activity.^27^ However, associations between brain GM and WM atrophy and cardiovascular fitness (as a proxy for physical activity) were observed even when activity levels were low (i.e. less than the recommended 150 minutes of moderate intensity activity a week).^30,31^ For people with MS who may not be able to achieve consistently high levels of activity (due to fatigue or fatiguability), identifying the role of intensity on the relationship between physical activity and brain structures could be transformative with regards to exercise recommendations and interventions for prevention and treatment.

#### Associations between disability scores

In our secondary analysis, the strong associations between STEPS and MS disability (EDSS) validate prior findings from our group and others.^13,16,32^ In a multivariable model, both STEPS and C2-3 TCA areas were significantly associated with EDSS, suggesting a potential contribution of TCA areas to EDSS not captured by STEPS.

When including STEPS as a covariate to predict EDSS values, cord white (and TCA) were retained in those models while cord GM was not. This suggests that the cord GM measured in this way does not explain significant additional variance in EDSS after adjusting for STEPS, further highlighting the strong relationship between cord GM and STEPS. However, cord WM seems to provide an additional relationship to EDSS not included in STEPS. This result will require further validation and investigation.

Walk-times (T25FW, TUG) were associated with upper cervical GM areas; if the decrease in upper cord area is believed to be generalizable for the whole cord,^4^ GM area (or change in area) could influence rhythmic, functional locomotion, potentially via central pattern generator (CPG) pathways (networks of spinal neurons).^33^ Walking involves coordinated movements between all four limbs. Excitatory inputs to the CPG at the upper (cervical, shoulder girdle) and lower (lumber, pelvic girdle) extremities are hypothesized to influence walking and reciprocal arm swing—which if damaged could impact physical ambulatory disability (and hypothetically decreased STEPS). Mechanisms for plasticity changes of CPG are not fully understood. However, indirect evidence from spinal cord injury models suggests there are adaptations and plasticity changes following injury at the cervical or thoracic spinal cord.^34^ To our knowledge, plasticity and reactivation of CPG in CNS demyelinating diseases (i.e. MS) have not been investigated; neither has the effect of changing physical activity levels. Increasing daily ambulation might prevent spinal cord GM atrophy, and therefore potentially slow disability progression (and improve walking). Longitudinal studies can determine the directionality of the associations and perhaps could identify avenues for physical/rehabilitative treatment interventions.

This study supports the potential value of STEPS as an indicator of dysfunction in systems other than ambulation. MS-specific symptoms (e.g. bowel and bladder disturbances, fatigue, pain) strongly correlated with STEPS, suggesting that remote physical activity monitoring provides additional information about overall MS disability and function beyond ambulatory volumetrics (i.e. the number/volume of steps taken per day). Correlations of STEPS with clinic-based and patient-reported outcomes were also comparable with those in earlier cohorts.^16,32,35^ These data are encouraging when considering opportunities for future interventions to improve symptoms and quality of life in people with MS—interventions that improve STEPS might also favorably impact historically difficult-to-treat symptoms such as fatigue and pain, or vice versa.^36^

Comorbidities or covariates of daily activity warrant specific attention, namely, mental health and body weight. With respect to mental health, previously reported associations between patient-reported mental health (mental health composite score, from the MS quality of life composite measure: MSQoL-29) and STEPS in a much larger (*N* = 492) international cohort of only progressive MS patients (SPI2, ρ = 0.130, *p* < 0.01)^16,32,35^ trended toward significance in this study. This might be expected given the smaller sample size and MS type (both relapsing and progressive) demographic. Although statistical significance was not reached regarding the greater variance in STEPS observed in people with lower BMI, future studies should consider including BMI, although it is possible that BMI does not have as strong or significant an association with physical activity in people with MS as observed in other populations.

This study had several limitations beyond the relatively small sample size. The cross-sectional design prevents identifying direction of causation between smaller spinal cord GM area and lower physical activity. Nevertheless, correlations between neuroanatomical, physiological disease effects, and activity are all consistent. Prospective, longitudinal data are needed to provide greater insight into the directionality of correlation between structural and physical functioning and disability worsening (changes). Longitudinal studies will also provide information regarding thresholds of significant physical activity levels that correspond with significant changes in structural pathology (MRI measures). Given the moderate disability (EDSS = 4.0) of our cohort due to the motor study inclusion criteria and the presence of both relapsing and progressive types of MS, generalizability to less impaired individuals with MS may be limited. Larger studies are needed to perform sub-analysis into specific disability levels within each MS type and level of disability.

## Conclusion

These results provide the first demonstration that spinal cord GM as an anatomical substrate associated with physical activity (STEPS). Longitudinal observations are needed to determine directionality and examine the value of STEPS as a proxy for generalized brain and cord volume loss. These results have potential implications for structural and functional modification of disease progression via therapeutic interventions aimed at altering STEPS.
